# BCL-3 loss sensitises colorectal cancer cells to DNA damage by targeting homologous recombination

**DOI:** 10.1016/j.dnarep.2022.103331

**Published:** 2022-07

**Authors:** Christopher Parker, Adam C. Chambers, Dustin J. Flanagan, Jasmine Wing Yu Ho, Tracey J. Collard, Greg Ngo, Duncan M. Baird, Penny Timms, Rhys G. Morgan, Owen J. Sansom, Ann C. Williams

**Affiliations:** a Colorectal Tumour Biology Group, School of Cellular and Molecular Medicine, Faculty of Life Sciences, Biomedical Sciences Building, University Walk, University of Bristol, Bristol BS8 1TD, UK; b Cancer Research UK Beatson Institute, Garscube Estate, Switchback Road, Bearsden, Glasgow G61 1BD UK; c Division of Cancer and Genetics, School of Medicine, Cardiff University, Cardiff CF14 4XN UK; d School of Life Sciences, University of Sussex, Sussex House, Falmer, Brighton BN1 9RH UK; e Biomedicine Discovery Institute, Monash University, Melbourne 3800, Australia

**Keywords:** Colorectal cancer/DNA Damage response**/**Therapy response**/**Chemoradiotherapy/Homologous recombination

## Abstract

The proto-oncogene BCL-3 is upregulated in a subset of colorectal cancers (CRC), where it has been shown to enhance tumour cell survival. However, although increased expression correlates with poor patient prognosis, the role of BCL-3 in determining therapeutic response remains largely unknown. In this study, we use combined approaches in multiple cell lines and pre-clinical mouse models to investigate the function of BCL-3 in the DNA damage response. We show that suppression of BCL-3 increases γH2AX foci formation and decreases homologous recombination in CRC cells, resulting in reduced RAD51 foci number and increased sensitivity to PARP inhibition. Importantly, a similar phenotype is seen in Bcl3^-/-^ mice, where Bcl3^-/-^ mouse crypts also exhibit sensitivity to DNA damage with increased γH2AX foci compared to wild type mice. Additionally, Apc.Kras-mutant x Bcl3^-/-^ mice are more sensitive to cisplatin chemotherapy compared to wild type mice. Taken together, our results identify BCL-3 as a regulator of the cellular response to DNA damage and suggests that elevated BCL-3 expression, as observed in CRC, could increase resistance of tumour cells to DNA damaging agents including radiotherapy. These findings offer a rationale for targeting BCL-3 in CRC as an adjunct to conventional therapies and suggest that BCL-3 expression in tumours could be a useful biomarker in stratification of rectal cancer patients for neo-adjuvant chemoradiotherapy.

## 1 Introduction

Rectal cancers constitute around 30% of colorectal cancer (CRUK). Whilst early-stage rectal cancers can be treated with surgical excision, advanced disease requires the addition of neo-adjuvant therapy to surgical resection to achieve cure. Neo-adjuvant therapy, such as studied in the Rapido [1] or PRODIGE 23 trials [2], reduces the risk of local or systemic recurrence in these high risk tumours. However, tumour response to neo-adjuvant therapy is variable [3] and understanding this variation is critical to improving outcomes in locally advanced rectal cancer.

Of the multitude of DNA lesions which result from exposure to DNA damaging therapeutics, the double strand break (DSB) is the most cytotoxic and thought to be the primary mode by which radiotherapy reduces tumour growth and survival [4]. DNA damage response (DDR) pathways are commonly aberrantly activated in cancer cells leading to tumour cell survival. There are two major DDR pathways by which DSBs can be repaired, non-homologous end joining (NHEJ) and homologous recombination (HR). NHEJ involves recognition of damage by KU proteins which activate DNA-PKcs, followed by repair of the break by end processing enzymes, DNA polymerases and DNA ligase IV [5]. This process is fast and active throughout the cell cycle but is error prone. In contrast, HR retains DNA sequence fidelity, using a sister chromatid as a template for repair, restricting the process to S and G2 phases of the cell cycle [6]). RAD51 and BRCA2 are critical to this process [7]. Dysregulation of these repair pathways is important in tumorigenesis but also confers targetable weaknesses within the tumour, such as by Poly ADP Ribose Polymerase (PARP) inhibitors [8].

BCL-3 is a proto-oncogene, highly expressed in a number of solid tumours [9]. It is an atypical member of the IκB family owing to its ability to activate or repress transcription of p50 and p52 NF-κB subunits [10]**.** Interactions between BCL-3 and co-regulatory proteins such as TIP60, JAB1, BARD1, PIR, HSP70, HDACs, CTBP1 and 2 and β-catenin [11], [12], [13], [14] have been observed, offering additional modes of transcriptional regulation by BCL-3. In CRC, BCL-3 was shown to promote cancer cell growth and survival by phosphoinositide 3-kinase (PI3K) and mammalian target of rapamycin (MTOR) mediated activation of the AKT pathway [15]. Additionally, it promotes colorectal cancer cell proliferation through regulation of Cyclin D1 [16] and stabilisation of c-MYC [17]. More recent work has demonstrated a further role for BCL-3 in cancer, promoting the stem cell phenotype in CRC, with implications for therapeutic resistance [14]. Clinically, BCL-3 expression is negatively correlated with survival in CRC patients, where strong nuclear staining of BCL-3 observed in tissue microarrays was associated with reduced patient survival [18] and additionally shown to be independent of tumour stage [14]. Given the poor outcome of patients with high BCL-3 expressing tumours and its previously characterised function in enhancing tumour cell survival, we hypothesised that BCL-3 expression may determine therapeutic resistance of tumours.

Here we present data identifying a novel role for BCL-3, where inhibiting BCL-3 expression sensitises CRC cell lines to irradiation-induced cell death by reducing HR and show that Bcl-3^-/-^ mice are sensitised to DNA damage inducing agents. This work furthers our understanding of therapeutic resistance in CRC and offers a rationale for targeting BCL-3 as an adjuvant to conventional therapies, particularly in the setting of neo-adjuvant therapy for locally advanced rectal cancer.

## 2 Materials and methods

### 2.1 Cell lines and cell culture

The human rectal carcinoma derived cell line SW1463, and colon carcinoma derived cell lines LS174T and LoVo were obtained from the American Type Culture Collection (ATCC), HCA7 (also colon cancer derived) cells were a kind gift from Sue Kirkham, Imperial College London. The U2OS human osteosarcoma derived cells were a kind gift from Dr Anna Chambers, University of Bristol. All cell lines were cultured in Dulbecco’s Modified Eagle’s Medium (DMEM, Sigma Aldrich, UK) supplemented with 10% foetal bovine serum (Sigma Aldrich, UK), 2 mM glutamine, 100 U/mL penicillin and 100 µg/mL streptomycin (Invitrogen). Cells were maintained at 37 °C in a dry incubator in 5% CO_2_.

### 2.2 Gene knockdown with RNAi

Cells were transiently transfected with small interfering RNA (siRNA, 50 nM, Dharmacon, UK) using Lipofectamine RNAi MAX (Thermo Fisher Scientific, Paisley, UK). BCL-3 was knocked down using a single siRNA sequence, confirmed with a second sequence (data not shown).

### 2.3 Generation of BCL-3 knockout cells with CRISPR-Cas9 ^D10A^ genome editing

CRISPR-Cas9 ^D10A^ based genome editing was performed according to the method developed by Chiang et al. [19] using the All-In-One vector, provided as a kind gift by Dr Paul Bishop, University of Bristol. Knockout of BCL-3 was confirmed by Sanger sequencing of edit site and absence of BCL-3 protein in western blot.

### 2.4 Irradiation

Cells were seeded 48 h prior to irradiation. Irradiation was performed with a Cs137 source in a RX30/55 M Irradiator (Gravatrom Industries Ltd.). For RAD51 foci assays, cells were treated with 3 µg/mL aphidicolin (Sigma-Aldrich, UK) immediately prior to irradiation to prevent progression from S-phase to G2 phase.

### 2.5 Tandem mass tagging (TMT) phosphoproteomics

HCA7 cells were lysed and 100 µg protein was trypsin digested (2.5 µg trypsin per 100 µg protein; 37 °C overnight) before being labelled with TMT Isobaric Mass Tag Labelling Kit (Thermo Fisher Scientific, UK). For phosphoproteomics, samples were enriched with a TiO2 based phosphopeptide enrichment kit (Pierce, USA). Samples were then pooled and fractionated by reversed phase liquid chromatography before analysis with nano-LC MSMS with an Orbitrap Fusion Tribrid mass spectrometer (Thermo Fisher Scientific, Paisley, UK). Phosphoproteomic TMT data was processed and quantified using Proteome Discoverer software v2.1 (Thermo Fisher Scientific, UK) and searched against the UniProt Human database and a Common Contaminants database using the SEQUEST algorithm. For phosphoproteomic analysis, phosphorylation as S, T and Y were also included as possible peptide modifications. All data was filtered to satisfy a false discovery rate (FDR) of 5%. Phosphopeptide abundances were ascertained and log2 transformed to linearise protein abundances. A two-tailed Student’s t-test was performed and proteins with a p-value ≤ 0.05 determined significant differences. The PANTHER (Protein Analysis Through Evolutionary Relationships) database was used to identify significantly enriched ontologies from the list of differentially expressed proteins. Further analysis was performed using Ingenuity Pathway Analysis (IPA, Qiagen, Manchester, UK). Fold changes and p-values were uploaded to IPA and a core analysis was performed to identify pathways that were significantly altered between different experimental conditions.

### 2.6 Crystal violet cell viability assay

Cells were seeded into and treated in 6 well plates and grown for 48 h before treatment. At 14 days after seeding, cells were fixed in 4% paraformaldehyde for 10 min and stained with 0.5% w/v crystal violet solution (Sigma-Aldrich, UK) for 10 min. Bound crystal violet was eluted with 500 µL 10% acetic acid and absorbance measured at 595 nm.

### 2.7 Immunofluorescence

Cells grown on glass coverslips were fixed with 4% paraformaldehyde for 10 min and permeabilized with 0.1% Triton-X 100. To prevent non-specific antibody binding, slides were blocked with 1% BSA. Cells were incubated with primary antibody at room temperature for one hour (phospho-histone H2A.X (1:10000, Millipore #05–636); RAD51(1:1000, Abcam); CENPF (1:500, Abcam #5) diluted in 1% BSA. After incubation with secondary antibody (Molecular Probes, Invitrogen, UK) the nuclei were stained 100 ng/µL DAPI. Coverslips were mounted with Mowiol 4–88 (Sigma-Aldrich, UK). Cells were imaged with a Leica DMI6000 inverted epifluorescence microscope with 100x lens and Photometrics Prime 95B sCMOS camera or a Leica SPE single channel confocal laser scanning microscope with 40x lens and Leica DFC365FX monochrome digital camera.

### 2.8 Immunoblotting

Whole cell lysates were made and subjected to SDS-PAGE/western blotting as previously described [20] using the following antibodies: BCL-3 (1:2000, Proteintech #, 23959,); CHK2 (1:1000, Millipore #05–649); phospho-CHK2 (1:1000, Cell Signalling Technology, #160); α Tubulin (1:10000, Sigma Aldrich, #T9026).

### 2.9 Clonogenic assay

1 × 10^4^ HCA7 cells were seeded per T25 flask and Olaparib (AZD2281, Selleckchem, UK) was added at indicated concentrations. After 12 days growth, cells were fixed with 10% neutral buffered formalin and stained with 1% Methylene blue (Sigma-Aldrich, UK) for counting number of colonies.

### 2.10 I-SceI DNA repair assay

On day one, U2OS reporter cell lines (kind gift from Prof J Stark, USA [21]) were transfected with siRNAs (40 nM) using lipofectamine RNAiMAX (Thermo Fisher Scientific) and transferred into new plates on day two. Cells were transfected on day three using Dharmafect kb (Dharmacon, UK) with a plasmid pCBASceI to induce I-SceI or with a transfection control plasmid mCherry2-C1. In addition, siRNAs (20 nM) were co-transfected to enhance knockdown of the target protein. Cells were analysed on day five using a LSRFortessa™ cell analyser (BD Biosciences, UK). The percentage of GFP positive cells were normalised with percentage of m-cherry positive cells to obtain the DNA repair efficiencies.

### 2.11 Mouse studies

All animal experiments were performed in accordance with UK Home Office regulations (under project licence 70/8646) and adherence to the ARRIVE guidelines and were subject to review by the Animal Welfare and Ethical Review Board of the University of Glasgow. All mice were maintained on a mixed C57BL/6 background. Mice were housed in conventional cages in an animal room at constant temperature (19–23 °C) and humidity (55% ± 10%) under a 12-h light–dark cycle and were allowed access to standard diet and water ad libitum. Mice of both genders, aged 2–6 months, were induced with a single intraperitoneal (i.p) injection of 2 mg tamoxifen (#T5648 Sigma-Aldrich,UK) as indicated. The transgenes/alleles used for this study were as follows: *Bcl3*^*-/-*^ [22], *VilCre*^ER^ [23] *Apc*^fl^ [24] and *Kras*^G12D^ [25]. For regeneration studies mice were given whole-body irradiation with γ-rays (10 Gy). For treatments, mice were dosed using the following regimens: Cisplatin (5 mg/kg, single intraperitoneal injection), Olaparib (50 mg/kg, once daily oral gavage) and Wee1 inhibitor ((Wee1i) 90 mg/kg, daily oral gavage). All of the listed drugs were made up in 0.5% Hydroxypropyl Methylcellulose (HPMC) and 0.1% Tween-80.

### 2.12 RNA in situ hybridisation

*In situ* hybridisation for *Lgr5* mRNA ((#311838, Advanced Cell Diagnostics) was performed using RNAscope 2.5 LS Reagent Kit–BROWN (Advanced Cell Diagnostics) on a BOND RX autostainer (Leica) according to the manufacturer’s instructions.

### 2.13 Mouse immunohistochemistry

Intestines were flushed with water, cut open longitudinally, pinned out onto silicone plates and fixed in 10% neutral buffered formalin overnight at 4 °C. Fixed tissue was rolled from proximal to distal end into swiss-rolls and processed for paraffin embedding. Tissue blocks were cut into 5 µm sections and stained with haematoxylin and eosin (H&E). Immunohistochemistry (IHC) was performed on formalin-fixed intestinal sections according to standard staining protocols. Primary antibodies used for IHC were against: BrdU (1:150, BD Biosciences, #347580), Rad51 (1:100, Abcam, #133534), Cleaved caspase 3 (1:500, Cell Signaling Technology, #9661) and H2ax (1:50, Cell Signaling Technology, #9718). Representative images are shown for each staining.

## 3 Results

### 3.1 BCL-3 depletion sensitises CRC cells to gamma irradiation

**Fig. 1:**
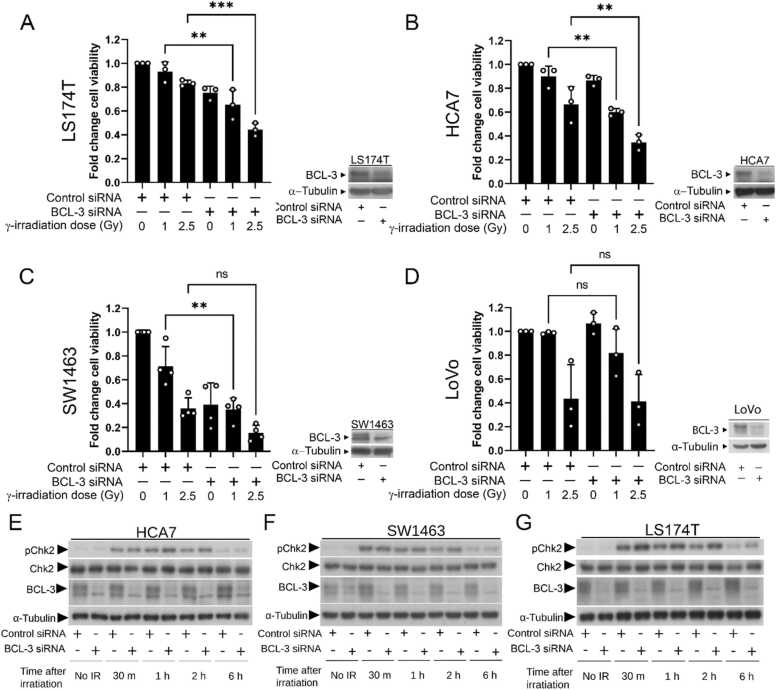
BCL-3 depletion increases sensitivity of colorectal cancer cell lines to gamma irradiation**.** Four colorectal cancer cell lines, LS174T (A), HCA7 (B), SW1463 (C) and LoVo (D) were treated with BCL-3 or control siRNA. 48 h later, cells were treated with indicated dose of gamma radiation. At 14 days after seeding, viable cells were stained with crystal violet and quantified by absorbance. Knockdown was confirmed by western blot. Data from three or four independent experiments, statistical significance determined using ANOVA. BCL-3 depletion increases ionising radiation induced downstream signalling. HCA7 (E), SW1463 (F) and LS174T (G) cells were treated with BCL-3 or control siRNA and given a 2.5 Gy dose of ionising radiation 48 h later. Cell lysates were taken at time points indicated and subjected to western blotting for total and phosphorylated CHK2 (Quantification of western blots is shown in Supplementary Figure 1). Tubulin was used as a loading control. Data representative of three experimental repeats.

To investigate whether targeting BCL-3 expression could increase the sensitivity of colorectal cell lines to gamma irradiation, four high BCL-3 expressing CRC cell lines (colon HCA7, LS174T, LoVo and rectal SW1463,) were transiently transfected with BCL-3 siRNA, seeded in a 6 well plate and irradiated 48 h later with gamma irradiation (1 and 2.5 Gy). After 14 days, viable cells were measured by crystal violet staining (Fig. 1, A-D). This revealed that in LS174T and HCA7 cells (Fig. 1A & B), BCL-3 knockdown caused lower viability at both 1 and 2.5 Gy, In the rectal SW1463 cells, (Fig. 1C), despite a significant increase in response to 1 Gy on knockdown of BCL-3, this was due to the loss of viability on suppression of BCL-3 expression and no additional effect with irradiation was detected. In contrast, in LoVo cells (Fig. 1D), there was no significant decrease in viability when BCL-3 was depleted with or without irradiation. These data suggest that, at least in a subset of CRCs, the presence of BCL-3 could be promoting radio-resistance enhancing tumour cell survival.

### 3.2 Loss of BCL-3 increases DSB associated signalling and DSB number after γ-irradiation in human colorectal cells

**Fig. 2:**
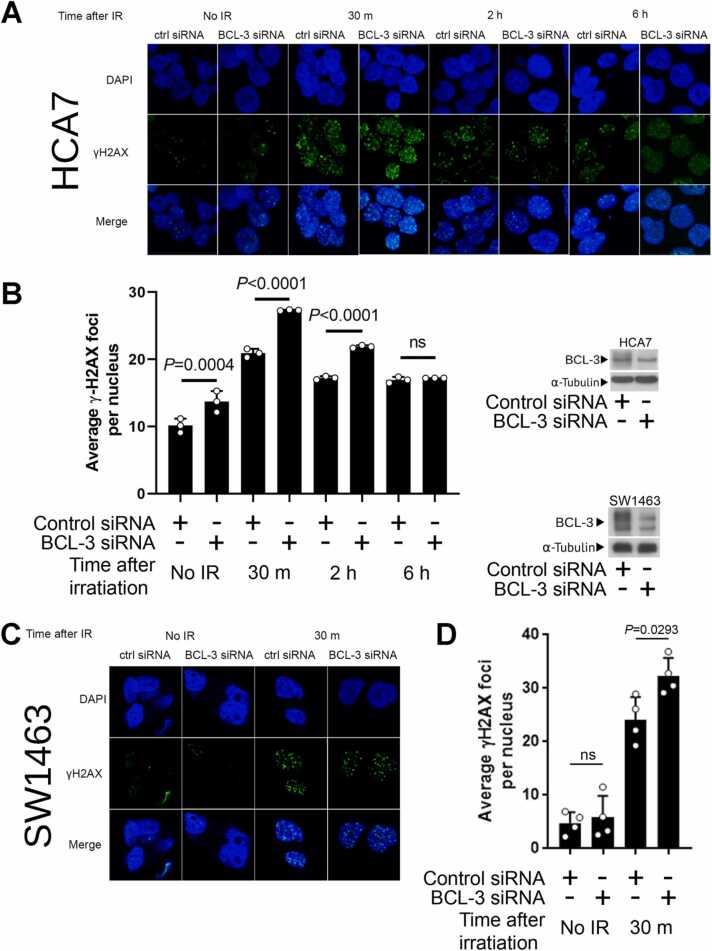
BCL-3 depletion increases ionising radiation induced double strand break number. HCA7 (A & B) and SW1463 (C & D) cells were treated with BCL-3 siRNA or control siRNA and given a 2.5 Gy dose of ionising radiation 48 h later. Cells were fixed at time points indicated after irradiation and stained for γ-H2AX (HCA7 A; SW1463 C). Quantification of average number of γH2AX foci per nucleus in HCA7 (B) and SW1463 (D) at timepoints indicated. Representative BCL-3 knockdown from these experiments was confirmed using western blot with tubulin used as a loading control for each cell line. Data from three independent experiments analysing > 30 nuclei per repeat. Statistical significance determined using ANOVA.

Because DSB generation is the primary mode of cell death/quiescence in irradiated cells, we hypothesised that suppression of BCL-3 expression reduced the capacity of the cell to repair DSBs. ATM is the primary transducer of signalling that communicates formation of DSBs from the proteins that recognise the break to effectors of repair [26]. After DNA damage, ATM is rapidly activated by autophosphorylation which initiates the phosphorylation and activation of a multitude of proteins involved in DNA repair and cell cycle arrest including H2AX and CHK2. To determine whether repression of BCL-3 changed signalling downstream of DSB formation, the sensitive CRC cells (LS174T, HCA7 and SW1464) were again transiently transfected with BCL-3 siRNA and treated with IR (2.5 Gy) 48 h later. Lysate samples were then taken between 0 and 6 h post irradiation. Western blotting was used to measure activation of CHK2 using phosphorylation specific antibodies (Fig. 1, E-G). In all three cell lines there was rapid induction of CHK2 phosphorylation after irradiation (within 30 min). When BCL-3 was suppressed in the cells, phosphorylation of CHK2 was further increased in the HCA7 cells at 30 min and 1 h after irradiation and in SW1463 and LS174T cells at 1 and 2 h post irradiation (refer to Supplementary figure 1), suggesting more damage had occurred following BCL-3 suppression. To investigate this further, foci of γ-H2AX, a marker that directly corresponds to DSB number, were quantified in HCA7 cells (chosen for amenability to imaging). BCL-3 was knocked down in HCA7 cell by transient siRNA transfection and cells were irradiated with 2.5 Gy, 48 h later (Fig. 2, A & B). Cells were fixed at points 0–6 h after irradiation and stained with antibody for visualisation of γ-H2AX. Interestingly, BCL-3 depleted HCA7 cells exhibited significant increased γ-H2AX foci number in the unirradiated cells (Fig. 2A & quantified in 2B), suggesting more DSB are present in the cells in which BCL-3 is suppressed. On induction of DSB using 2.5 Gy γ-irradiation, this difference was further increased, with the BCL-3 siRNA transfected cells having significantly higher levels of γ-H2AX foci for up to two hours after irradiation. This increase in DSB number suggests that depletion of BCL-3 increases spontaneous break formation and/or reduces rate of DSB repair and supports data in Fig. 1 showing an increased sensitivity to irradiation following BCL-3 suppression [27]. By six hours after irradiation the difference in γ-H2AX foci number is lost, with the number of γ-H2AX foci in BCL-3 siRNA being similar to that of the siRNA control cells at the two-hour time point (Fig. 2B). This result suggests that either there is more damage which is repaired within 6 h, or reduced repair at the early timepoints. The increase in γH2AX foci formation following BCL-3 suppression and irradiation was confirmed in the rectal cancer SW1463 cells 30 min after irradiation (Fig. 2C & quantified in 2D).

### 3.3 Phosphoproteomics reveal a role for BCL-3 in the DNA damage response

**Fig. 3:**
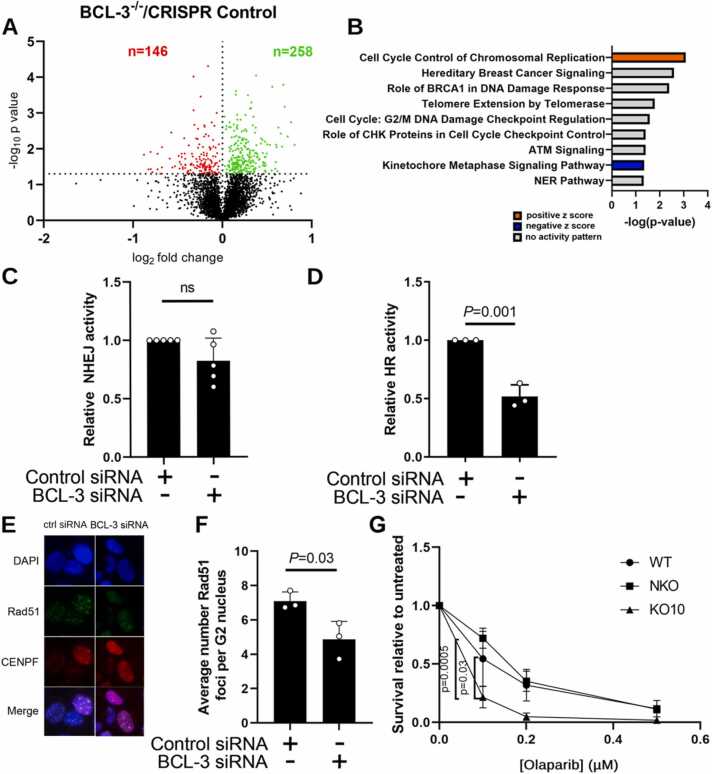
BCL-3 knockdown reduces rate of homologous recombination in U2OS and colorectal cancer cells. (A) Volcano plot of differentially phosphorylated proteins from phosphoproteome of *BCL-3*^-/-^ vs CRISPR control HCA7 cells. Increased abundance of 258 phosphopeptides (green) and decreased abundance of 146 phosphopeptides (red). Horizontal dashed line indicates significance at -log_10_ p-value = 1.30 (B) Canonical pathway enrichment analysis using Ingenuity Pathway Analysis (IPA) of differentially phosphorylated proteins when comparing *BCL-3*^-/-^ vs CRISPR control HCA7 cells. Level of enrichment is shown by -log(p-value). Z-score indicates predicted activation state of the pathway shown. Blue indicates a negative z-score and pathway inhibition. Orange indicates a positive z-score and pathway activation. Grey indicates significant change in pathway without predicted direction. BCL-3 was knocked down in EJ5-GFP (C) and DR-GFP (D) U2OS cells transfected with a I-SceI expression plasmid. NHEJ (C) and HR (D) activity were measured by percentage of GFP positive cells in FACS, expressed relative to control siRNA population. Significance measured with two tailed T-test. (E) U2OS cells were fixed two hours after 2.5 Gy irradiation in cells treated with indicated siRNA. RAD51, CENPF and DAPI were stained for and confocal microscopy performed (exemplary images shown). (F) Quantification of RAD51 foci in CENPF positive cells shown in (E). Data are mean of three independent experimental repeats ± s.d. Significance measured by two tailed T-test. (**G**) WT, CRISPR control (NKO) and BCL-3 knock out (KO) cells were seeded at equal density and treated six hours later with olaparib. Number of colonies formed was counted two weeks after seeding, represented as relative mean survival of three independent experimental repeats ± s.d. Statistical significance measured by one way ANOVA.

Having observed an increase in DSBs in cells with reduced BCL-3 expression, we sought to investigate the possible mechanism. To take an unbiased view of potential pathways changed following loss of BCL-3, TMT-phosphoproteomics was performed on whole cell HCA7 lysates. For these experiments BCL-3 was deleted by CRISPR-Cas9^D10A^ gene editing [19], resulting in production of a BCL-3 knockout clone (KO) and a pool of control cells (NKO) which had undergone the knockout protocol but in which BCL-3 was not deleted (see Supplementary Figure 2). Wildtype (WT), NKO and KO HCA7 cells were seeded at equal density and lysates were taken after 48 h of growth. Lysates were trypsin digested and the resulting peptides differentially labelled prior to MS/MS analysis. The data produced were subject to a Sequest search against the Uniprot human database and filtered at a 5% false discovery rate. Protein phosphorylation that was affected by BCL-3 deletion was identified as that with significantly different (p < 0.05) abundance between the NKO and BCL-3 KO populations after excluding those proteins that also changed between WT and NKO cells (Fig. 3A). This showed an increased abundance of 258 phosphopeptides and decreased abundance of 146 phosphopeptides in the BCL-3 KO cells relative to the NKO cells (Fig. 3A). Ingenuity Pathway Analysis (IPA, QIAGEN) revealed that a number of pathways related to DNA damage repair (DDR) and control were changed by the loss of BCL-3 (Fig. 3B). Of interest, changes in “the role of BRCA1 in DNA Damage Response” canonical pathway suggest that phosphorylation of proteins directly involved in DNA damage repair could be changed in BCL-3 KO cells. This includes DNA repair proteins RAD50, RFC1 and FANCD2. Examining changes in biological functions by IPA showed a predicted increase in DNA damage in the BCL-3 KO cells relative to the NKO cells. This analysis was repeated in another BCL-3 KO colorectal cell line, demonstrating similar changes in pathways involved in homologous recombination (Supplementary Figure 3).

### 3.4 BCL-3 promotes DSB repair by homologous recombination

To investigate whether loss of BCL-3 expression regulated DSB repair in cells, we used EJ5-GFP and DR-GFP assays to measure the effects of BCL-3 depletion on rates of NHEJ and HR respectively [21]. These assays use U2OS cell lines with an integrated cassette for GFP, the sequence of which is interrupted by a restriction site for the restriction enzyme I-SceI. Expression of I-SceI in these cells generates double strand breaks which can be repaired only by the pathway specific to the cell line (HR; DR-GFP or NHEJ; EJ5-GFP), generating GFP+ cells that are subsequently quantified by flow cytometry (Supplementary Figure 4A & B). BCL-3 was depleted in the U2OS EJ5-GFP cells by siRNA transfection, followed 48 h later by I-SceI transfection. Flow cytometry subsequently revealed that in there was no significant difference in reporter activity (Fig. 3C) suggesting that changes in BCL-3 expression did not affect NHEJ. However, there was a marked and significant decrease in reporter activity in DR-GFP cells when BCL-3 was suppressed (Fig. 3D), consistent with a drop in HR and in concordance with HR being the most changed pathway in the phosphoproteome.

RAD51 is a HR specific repair factor that accumulates at repair foci in S and G2 phase cells. To further investigate whether suppression of BCL-3 reduced HR as shown by the DR-GFP reporter assay, quantitation of RAD51 foci by immunofluorescence in U2OS cells was used as a measure of HR activity [28]. This cell line was selected for its suitability in immunofluorescent imaging of RAD51 after unsuccessful attempts to stain RAD51 in HCA7 cells. To restrict the analysis to cells in which HR is active, cells were co-stained with CENP-F, a marker of G2 and S phase cells. BCL-3 was knocked down in U2OS cells by transient siRNA transfection (Supplementary Figure 4C) and the cells were irradiated with 2.5 Gy 48 h later. The cells were fixed two hours after irradiation to capture the peak in foci number [29] and RAD51 foci were visualised with immunofluorescence using a recombinant RAD51 antibody (Fig. 3E). Foci were counted in a minimum of 60 CENP-F positive nuclei per experimental repeat; the number of RAD51 foci were significantly reduced in BCL-3 depleted U2OS cells (Fig. 3F), supporting the role of BCL-3 in HR.

### 3.5 BCL-3 depletion sensitises CRC Cells to PARP inhibitors

Cells with a defect in any component of HR are sensitive to treatment with PARP inhibitors, as exploited therapeutically in cancers where BRCA1 or BRCA2 are mutated [30]. Inhibition of PARP reduces SSB repair resulting in a greater DSB load when SSBs are converted to DSBs in DNA replication [30]. The effect of BCL-3 on sensitivity to PARP inhibition was assessed by clonogenic assay where WT, NKO and BCL-3 KO HCA7 were seeded into flasks at 1 × 10^4^ per flask and treated with olaparib. After 14 days, the number of colonies formed was counted. BCL-3 KO CRC cells were more sensitive to the PARP inhibitor olaparib than control cells, showing reduced colony formation after treatment (Fig. 3G), concordant with defective HR in the CRC cells.

### 3.6 BCL-3 protects the intestinal crypt from DNA damage

**Fig. 4:**
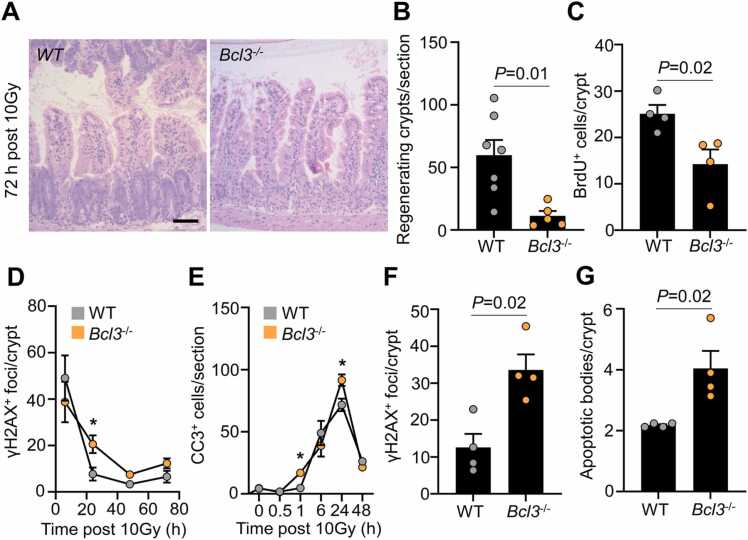
Loss of Bcl3 impairs intestinal regeneration and sensitises the epithelium to DNA damage. (A) Representative H&E staining of wild-type (WT) and Bcl3KO (*Bcl3*^-/-^) mice 72 h following whole-body irradiation (10 Gy). Scale bar, 100 µm. (B) Quantification of regenerating crypts per intestinal cross section from mice described in A. n = 7 WT, n = 5 *Bcl3*^-/-^. Data are ± s.e.m; Mann–Whitney two-tailed U-test. (C) Quantification of BrdU^+^ cells per regenerating crypt from mice described in A. n = 4 WT, n = 4 *Bcl3*^-/-^. Data are ± s.e.m; Mann–Whitney two-tailed U-test. (D) Quantification of γH2AX^+^ foci per crypt over time following whole-body irradiation (10 Gy). n = 4 WT, n = 4 *Bcl3*^-/-^. Data are ± s.e.m; *P = 0.01. Mann–Whitney two-tailed U-test. (E) Quantification of cleaved caspase-3^+^ (CC3^+^) cells per intestinal cross section over time following whole-body irradiation (10 Gy). n = 4 WT, n = 4 *Bcl3*^-/-^. Data are ± s.e.m; *P = 0.01. Mann–Whitney two-tailed U-test. (F) Quantification of γH2AX^+^ foci per crypt 24 h following single dose of cisplatin (5 mg/kg) in mice of the indicated genotypes. n = 4 WT, n = 4 *Bcl3*^-/-^. Data are ± s.e.m; Mann–Whitney two-tailed U-test. (G) Quantification of apoptotic bodies per crypt in mice described in F. n = 4 WT, n = 4 *Bcl3*^-/-^. Data are ± s.e.m; Mann–Whitney two-tailed U-test.

To translate our findings to a more complex system, we used BCL-3 knockout mice (*Bcl3*^-/-^) to examine the sensitivity and response of the intestinal epithelium following ionising radiation and DNA damage. In this context, wild-type control littermates (WT) and *Bcl3*^-/-^ mice were given a dose of whole body γ-irradiation (10 Gy), triggering a well-defined cascade of cell death (6–24 h) and epithelial regeneration (72 h) following damage [31]. Compared to WT control, the regenerative capacity of *Bcl3*^-/-^ mice was significantly impaired, as measured by the total number of regenerating crypts and BrdU^+^ crypt cells (Fig. 4A-C). Given the increased frequency of DSBs following loss of BCL-3 in CRC cells, we sought to determine whether DSBs are also increased in *Bcl3*^-/-^ intestinal epithelia. In line with previous results (Fig. 2), *Bcl3*^-/-^ intestinal crypts showed higher levels of γH2AX foci 24 h post-irradiation compared to control mice (Fig. 4D). Importantly, *Bcl3*^-/-^ mice showed increased cleaved-caspase 3^+^ (CC3^+^) cells at the same time point (24 h) compared to control (Fig. 4E), suggesting that failure to repair DSBs triggers cell death, and subsequently compromised tissue regeneration. Importantly, cells with defective DDR or deficient HR show sensitivity to drugs that induce DNA damage, such as cisplatin [30], [32]. We leveraged this, and treated *Bcl3*^-/-^ mice with cisplatin (5 mg/kg), olaparib (50 mg/kg) or a wee1 inhibitor (90 mg/kg) for 5 days to better understand the function of BCL-3 in regulating DNA damage response.

**Fig. 5:**
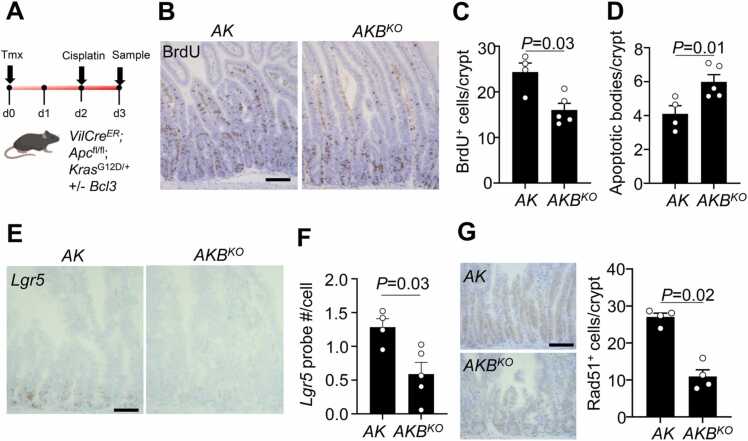
Loss of Bcl3 sensitises mouse models of CRC to DNA damage. (A) Schematic illustrating tamoxifen induction, treatment regimen and tissue harvest of *VilCre*^*ER*^*;Apc*^fl/fl^*;Kras*^G12D/+^ mice + /- *Bcl3*. (B) Representative BrdU staining of tamoxifen-induced *VilCre*^*ER*^*;Apc*^fl/fl^*;Kras*^G12D/+^ (*AK*) and *VilCre*^*ER*^*;Apc*^fl/fl^*;Kras*^G12D/+^;*Bcl3*^-/-^ (*AKB*^*KO*^) mice 24 h following cisplatin treatment (5 mg/kg). Scale bar, 100 µm. (C) Quantification of BrdU^+^ cells per crypt/villus unit in mice described in B; n = 4 *AK*, n = 5 *AKB*^*KO*^. Data are ± s.e.m; Mann–Whitney two-tailed U-test. (D) Quantification of apoptotic bodies per crypt in mice described in B; n = 4 *AK*, n = 5 *AKB*^*KO*^. Data are ± s.e.m; Mann–Whitney two-tailed U-test. (E) Representative *Lgr5* (in-situ hybridisation) staining of mice described in B. Scale bar, 100 µm with quantification of staining as total probe copy number by total cell count. (F) Quantification of Lgr5 staining as total probe copy number by total cell count. N = 4 *AK*, n = 5 *AKB*^*KO*^. Data are s.e.m; Mann-Whitney two-tailed U-test. (G) Representative staining (left) and quantification (right) of Rad51^+^ cells in mice described in B. Scale bar, 100 µm. n = 4 *AK*, n = 4 *AKB*^*KO*^. Data are ± s.e.m; Mann–Whitney two-tailed U-test.

Examination of crypts 24 h following treatments showed that there was no significant difference in number of BrdU^+^ cells between WT and *Bcl3*^-/-^ mice with any treatment (Supplementary Figure 5A). Although these results may initially appear at odds with the decrease in BrdU+ cells observed in *Bcl3*^-/-^ mice following irradiation, this is consistent with previous findings. The intestine is one of the most radio-sensitive tissues in the body and the response to whole body irradiation (10 Gy) is different to those observed following 5 mg/kg cisplatin or 50 mg/kg olaparib [33], [34]. One important difference is widespread epithelial denudation following whole-body irradiation, where the villus and crypt epithelium are shed from the submucosa. As a consequence, any impairment in regeneration (which requires the assembly and mobilisation of stem cell populations), will be evident via changes in cell proliferation. The dose of cisplatin used in the current study, and to a lesser extent olaparib, do not evoke the same extent of epithelial shedding, and therefore do not necessarily trigger large differences in cell proliferation. Importantly, both cisplatin and olaparib are effective inducers of DNA damage and DSBs, which was observed and quantified in WT and *Bcl3*^-/-^ mice (Fig. 4F). Importantly, we demonstrate a significant increase in γH2AX foci and apoptotic bodies in *Bcl3*^-/-^ mice compared to WT when treated with cisplatin (Fig. 4F & G). Similarly, *Bcl3*^-/-^ mice treated with olaparib had increased γH2AX foci compared to WT control (Supplementary Figure 5B), consistent with findings in the CRC cells (Fig. 2, Fig. 3). Interestingly, there was no change to DNA damage (γH2AX foci) or cell death following wee1 inhibition (Supplementary. Fig. 5B & C), which is consistent with its non-HR targeted mechanism of action [35]. Taken together, the increased damage seen in *Bcl3*^-/-^ mice with cisplatin and in particular, olaparib treatment is consistent with a defect in HR as seen in BCL3-deficient human CRC cells.

### 3.7 Loss of Bcl3 sensitises mouse models of CRC to DNA damage

To extend and confirm our findings in human CRC cells, we generated mice harbouring common CRC mutations, *Apc* and *Kras*, targeted to the intestinal epithelium (*VilCre*^ER^;*Apc*^fl/fl^;*Kras*^G12D/+^, hereafter *AK*) and crossed them to *Bcl3*^-/-^ mice (*VilCre*^ER^;*Apc*^fl/fl^;*Kras*^G12D/+^;*Bcl3*^-/-^, hereafter *AKB*^*KO*^). Both *AK* and *AKB*^*KO*^ mice were induced with tamoxifen (2 mg) and treated with a single dose of cisplatin (5 mg/kg) and harvested 24 h after (Fig. 5A). Consistent with cisplatin’s mechanism of action [36], highly-proliferative transit-amplifying cells were significantly reduced in both *AK* and *AKB*^*KO*^ mice compared to untreated controls (Fig. 5B, Supplementary Figure 5D). Compared to cisplatin-treated *AK* mice, cisplatin-treated *AKB*^*KO*^ mice had reduced BrdU^+^ crypt cells with a concomitant increase in apoptosis (Fig. 5B-D, Supplementary Figure 5D & E), indicating loss of Bcl3 sensitises *Apc.Kras*-mutant cells to cisplatin.

We have previously showed Bcl3 promotes stem-cell phenotypes in CRC cells [14], and therefore wanted to determine whether the combination of DNA damage (cisplatin) and Bcl3 deficiency preferentially targets *Lgr5*^+^ intestinal stem cells [37] and/or transit-amplifying progenitor cells. In support of our previous work [14], *Lgr5* expression was markedly reduced in cisplatin-treated *AKB*^*KO*^ mice, while *AK* mice retained *Lgr5* expression, suggesting cisplatin and loss of Bcl3 synergise to eliminate both the TA and importantly the Lgr5^+^ ISC populations (Fig. 5E & F). Finally, to investigate whether the increased sensitivity following cisplatin in *AKB*^*KO*^ mice was due to defective HR, we quantified Rad51^+^ crypt cells following cisplatin. Consistent with previous results (Fig. 3E & F), *AKB*^*KO*^ mice had reduced Rad51^+^ cells following DNA damage (cisplatin) compared to *AK* mice (Fig. 5G), indicating Bcl3 is required for optimal HR signalling after DNA damage.

## 4 Discussion

Given the relationship between BCL-3 expression levels in CRC and poor patient prognosis [14], [18], we sought to investigate whether BCL-3 plays a role here by altering tumour response to therapeutic agents. Here, we report that loss of BCL-3 sensitises colorectal cells to DNA damaging therapeutic agents both in human CRC cells and in mice. Although BCL-3 has been studied in the context of DNA damaging agents such as UV irradiation [38], [39], [40] and alkylating agents [41], to our knowledge no investigation have been made of a role for BCL-3 in the DDR. This builds on previous work that has characterised a role for BCL-3 in promoting tumour survival, inhibiting apoptosis through the AKT pathway [15], HDM2 [39], [42], [43] and DNA-PK [39]. In addition, recent work in our group has also demonstrated BCL-3 promoting the stem cell phenotype in CRC cells, a phenotype associated with therapeutic resistance [14]. Here, we observed increased γH2AX foci number upon BCL-3 loss in both CRC cells and mouse epithelium after irradiation, suggesting that this effect is the result of either a greater amount of DNA damage being sustained or that cells with depleted BCL-3 have acquired a defect in the DDR. The observed reduction in HR activity coupled with increased sensitivity to PARP inhibition in BCL-3 depleted cells, both in human CRC cells and in mice, suggests that loss of repair activity is at least in part responsible for greater DSB number.

Several earlier studies could potentially shed light on mechanisms by which BCL-3 may regulate the rate of DNA repair by HR. BCL-3 is thought to bind to a number of proteins involved in DSB repair. Dechend et al. [11] identified interaction of a BCL-3-p50 complex with JAB1, BARD1 and TIP60. BARD1 is the major binding partner of BRCA1 [44] which in turn plays a direct role in HR, directing RAD51 filament formation and inhibiting NHEJ [6]. The upregulation of this pathway with loss of BCL-3 seen in phosphoproteomics in BCL-3 KO cells could be to compensate the loss of the interaction between BCL-3 and BARD1. TIP60 is a histone acetyl transferase (HAT) and another key DNA repair protein, required for acetylation and activation of ATM and DNA-PKcs in addition to a role in acetylation of histones H2AX and H4 at DSBs [45]. BCL-3 also interacts with the HAT p300/CBP[46] and histone deacetylases (HDACs)[12], [47], [48], [49]. HDACs are commonly upregulated in CRC and these proteins are required for expression and catalytic activity of a multitude of DNA repair proteins as well as altering chromatin compaction for repair [50]. Therefore, loss of BCL-3 could be mimicking the radiation sensitivity seen upon knockdown of class I HDACs [51], [52].

The identification of BCL-3 as a HR promoting factor has several implications for treatment of CRC patients. The observed increase in radiosensitivity upon BCL-3 depletion suggests that rectal patients who have higher tumour BCL-3 expression would be less likely to respond to neo-adjuvant radiotherapy, therefore stratification of therapy by BCL-3 expression could avoid unnecessary treatment or necessitate the addition of adjunctive therapies to improve therapeutic response [53]. However, we observed that sensitisation to γ-irradiation by BCL-3 knockdown was not universal across the high BCL-3 expressing cell lines tested, a comparison between BCL-3 expression in patient tissue and response to radiotherapy will therefore be required to demonstrate the applicability of BCL-3 as a predictor of therapeutic response.

Adjuvant treatment with novel chemical inhibitors of BCL-3 [54] may allow patients with higher BCL-3 expression to be sensitised to conventional radiotherapy, improving therapeutic response. As demonstrated in vivo, this finding would also be relevant to DNA damaging chemotherapy. Several agents that cause DNA damage repaired by HR are used or have been trialled in CRC. As well as platinum based compounds, Irinotecan targets topoisomerase I during DNA replication, causing DSBs [55] whilst Veliparib is a PARP inhibitor and has been used in trials in combination with other drugs [56]. Furthermore, there is increasing interest in the concept of BRCAness where factors other than BRCA1 or BRCA2 mutation result in defective HR and an expanding group of proteins are being targeted to increase sensitivity to agents such as PARP inhibitors [57]. Our results suggest that targeting BCL-3 during treatment to achieve an HR reduced phenotype would increase the efficacy of these drugs. Alternatively, BCL-3 expression could be used to stratify patients for treatment with these drugs, avoiding their use in more HR proficient cells. Excitingly, given the expression of BCL-3 in a wide range of cancers including breast cancer, these findings could translate into improving therapy response or stratifying treatment in other cancer types beside CRC.

## 5 Conclusions

In conclusion, we have demonstrated a novel role for BCL-3 in conferring resistance to IR in colorectal cancer cells and in mouse intestine. In addition, we have shown that inhibition of BCL-3 function could increase the therapeutic response to chemotherapeutic drugs as well. We propose that patients with higher BCL-3 expressing colorectal tumours have poorer survival at least in part because it promotes increased HR activity, allowing cells to tolerate and repair DNA damage caused by therapeutic agents.

## Ethics approval and consent to participate

All animal experiments were performed in accordance with UK Home Office regulations (under project licence 70/8646) and adherence to the ARRIVE guidelines and were subject to review by the Animal Welfare and Ethical Review Board of the University of Glasgow.

## Conflicts of Interest

The authors declare that there are no conflicts of interest.

## Funding

CP was supported by a Wellcome Ph.D. studentship (203988/Z/16/Z); ACC by a Medical Research Council Clinical Research Training Fellowship (MR/N001494/1); DF, TJC, OJS and ACW by an MRC Research Grant (MR/R017247/1); GN and DMB by Cancer Research UK programme grant (A18246/A29202); PT by a Ph.D. Studentship from Bowel & Cancer Research; RGM by a Kay Kendall Leukemia Fund (10.13039/501100000402KKLF) Junior Fellowship (KKL1051), CP, ACC, PT by the John James Bristol Foundation. The funding sources had no involvement in study design; collection, analysis or interpretation of data; writing the report.

## CRediT authorship contribution statement

AC, DMB, OJS, ACW – Conceptualization; CP, ACC, TJC, GN, RGM, DJF, DMB, OJS, ACW. CP, ACC, TJC, PT and RGM - in-vitro data curation and formal analysis. GN and DMB - in-vitro data curation and formal analysis of I-Scel assay. DJF, JWYH and OJS - in-vivo methodology, data curation and formal analysis; CP, ACC, DJF and GN -Visualisation; All authors read, reviewed and edited original daft and final manuscript.

## Data Availability

Data generated or analysed during this study are included in this published article [and its Supplementary information files], with any datasets used and/or analysed during the current study are available from the corresponding author on reasonable request.
