# Corrigendum to “BCL-3 loss sensitises colorectal cancer cells to DNA damage by targeting homologous recombination” [DNA Repair 115 (2022) 103331]

**DOI:** 10.1016/j.dnarep.2024.103800

**Published:** 2025-01

**Authors:** Christopher Parker, Adam C. Chambers, Dustin J. Flanagan, Jasmine Wing Yu Ho, Tracey J. Collard, Greg Ngo, Duncan M. Baird, Penny Timms, Rhys G. Morgan, Owen J. Sansom, Ann C. Williams

**Affiliations:** a Colorectal Tumour Biology Group, School of Cellular and Molecular Medicine, Faculty of Life Sciences, Biomedical Sciences Building, University Walk, University of Bristol, Bristol BS8 1TD, UK; b Cancer Research UK Beatson Institute, Garscube Estate, Switchback Road, Bearsden, Glasgow G61 1BD, UK; c Division of Cancer and Genetics, School of Medicine, Cardiff University, Cardiff CF14 4XN, UK; d School of Life Sciences, University of Sussex, Sussex House, Falmer, Brighton BN1 9RH, UK; e Biomedicine Discovery Institute, Monash University, Melbourne 3800, Australia

The authors regret that the Ref. [22] is wrong. The correct reference is S. Paxian, H. Merkle, M. Riemann, M. Wilda, G. Adler, H. Hameister, S. Liptay, K. Pfeffer, R.M. Schmid. Abnormal organogenesis of Peyer's patches in mice deficient for NF-kappaB1, NF-kappaB2, and Bcl-3. Gastroenterology, 122(7), 2022, pp. 1853–68. doi: 10.1053/gast.2002.33651. PMID: 12055593.

The authors would like to apologise for any inconvenience caused.

