# Supplementary material for: BCL-3 loss sensitises colorectal cancer cells to DNA damage by targeting homologous recombination
Source: DNA Repair (Amst). 2022 Jul;115:103331. doi: 10.1016/j.dnarep.2022.103331 (PMC10618080; doi:10.1016/j.dnarep.2022.103331)
Supplement: Supplementary file 1 — Supplementary material [file mmc1.docx]

**Supplementary Figure 1**

**
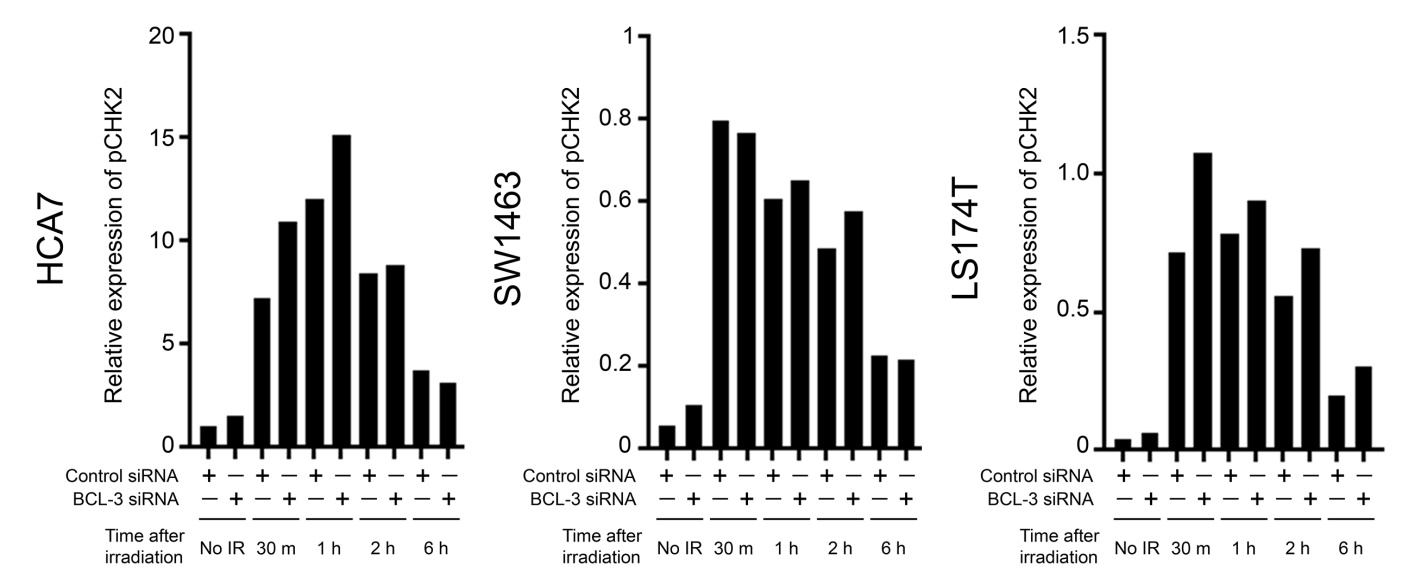
**

**Supplementary Figure 1** Quantification of western blots found in Figure 1. Image J was used to quantify pCHEK2 expression with normalisation to Tubulin loading controls. BCL-3 knockdown using siRNA is observed to increase expression of pCHK2. Data is exemplary data for n=3 independent experiments.

**Supplementary Figure 2**


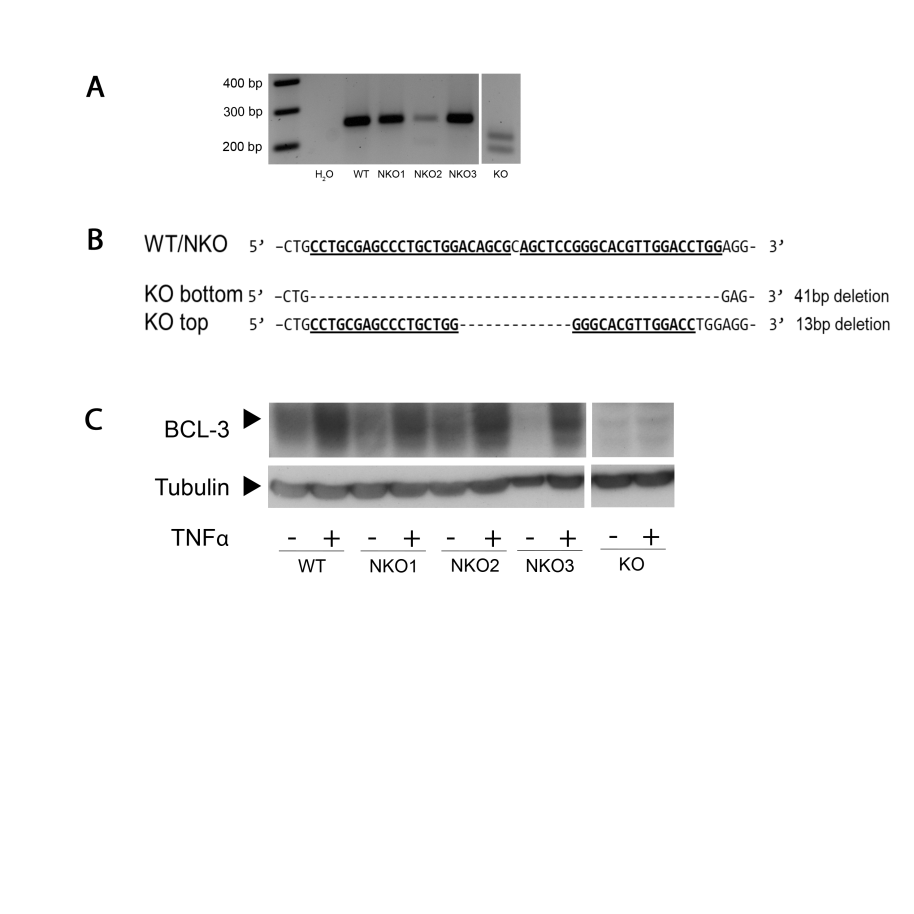


**A**

**C**

**B**

**Supplementary Figure 2** **A**, DNA extracted from HCA7 clones [WT; non- knockout control, (NKO) and knockout, (KO)) after the BCL-3 knockout process underwent PCR with primers complementary to the Cas9^D10A^ target site. PCR products were subsequently separated on a 2% agarose gel by electrophoresis. **B**, PCR products underwent Sanger sequencing demonstrating identical sequences in the WT cells and NKO clones and deletions of 41 base pairs and 13 base pairs in the bottom and top bands of the KO clone. Sequence shown flanks the Cas9^D10A^ target site with sgRNA sequences shown in bold underlined. **C**) BCL-3 expression from WT, NKO and KO clones after TNFα stimulation confirming loss of BCL-3 expression in the KO clone.

**Supplementary Figure 3**

**
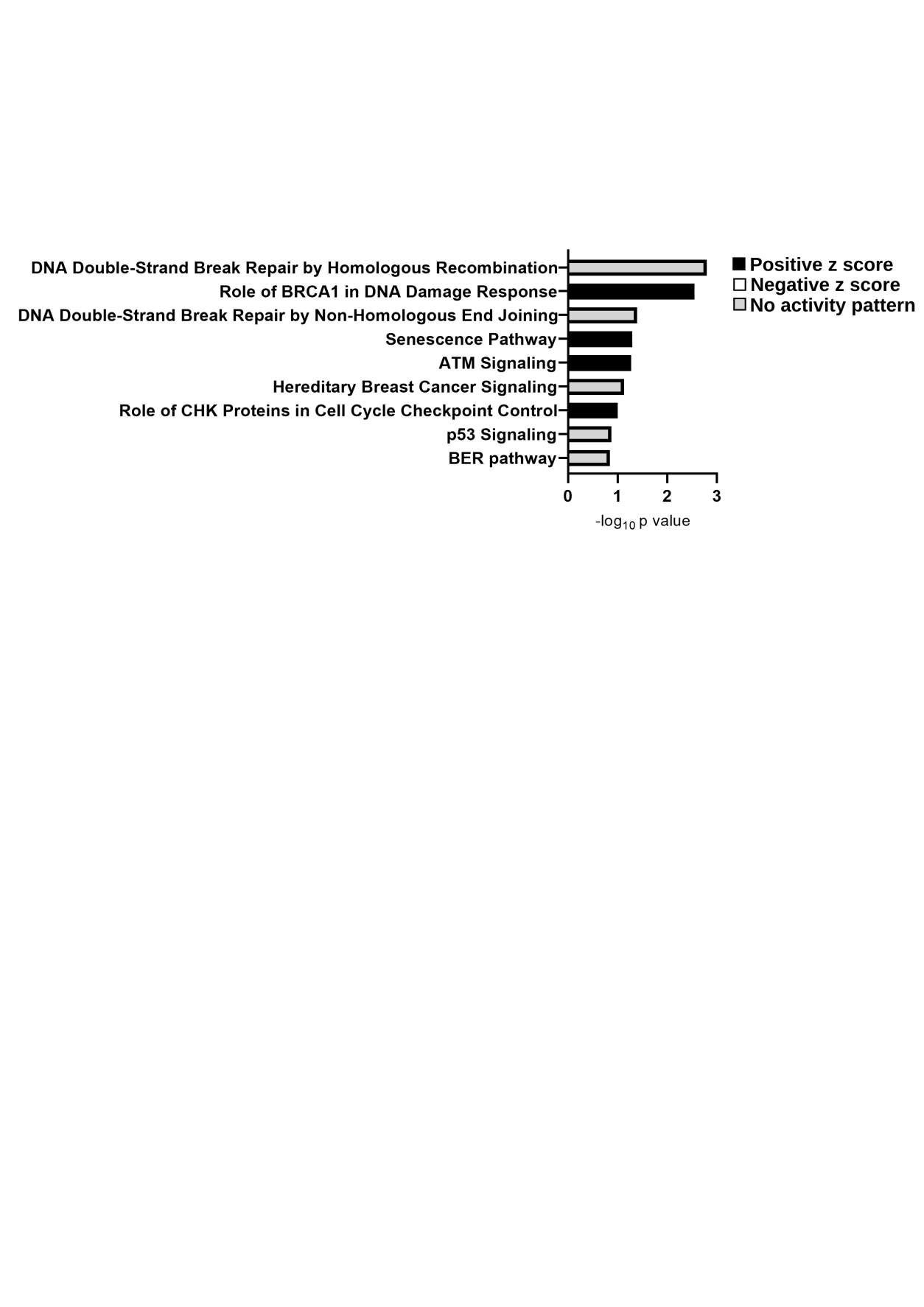
**

**Supplementary Figure 3**. Canonical pathway enrichment analysis of differentially expressed phosphoproteins when comparing BCL-3 KO vs CRISPR NKO control in SW620 human CRC cells. Level of enrichment is shown by -log(p-value). Z-score indicates the of predicted activation state of the canonical pathway. Blue indicates a negative z-score and pathway inhibition. Orange indicates a positive z-score and pathway activation.

**Supplementary Figure 4**


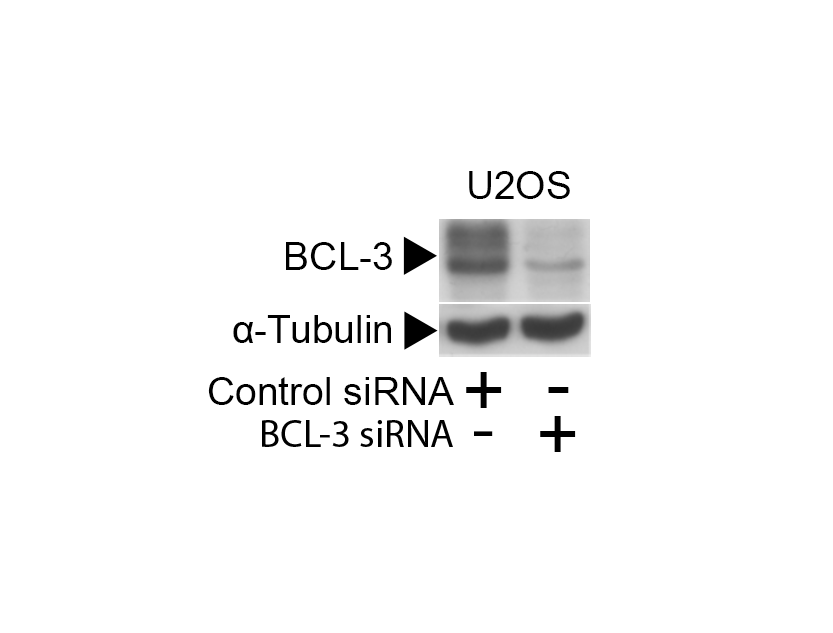

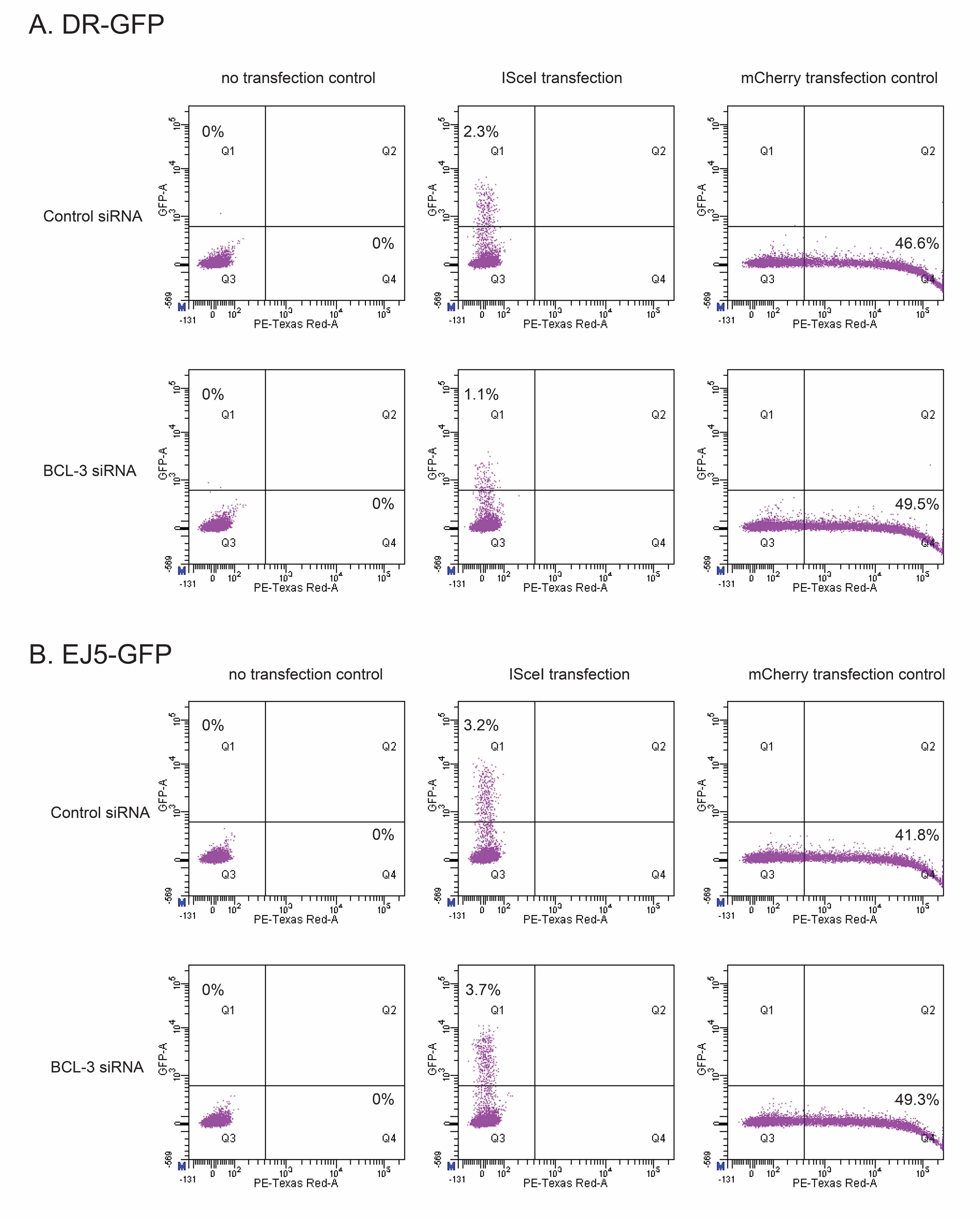


C.

**Supplementary Figure 4** U2OS cells were co-transfected with siRNA targeting BCL-3 or Control as well as with the I-SceI or Control mCherry expression plasmid. HR **A** and NHEJ **B** activity were measured by percentage of GFP/mCherry positive cells using FACS showing no significant difference in transfection efficiency. Data shown is flow cytometry data from one representative experiment. **C** U2OS cells were transfected with Control or BCL-3 siRNA. Cells were irradiated with 2.5 Gy at 48 h following transfection, then fixed 2 h after irradiation, with immunofluorescence performed for RAD51, CENP-F and DAPI (see Figure 3); protein lysates were also collected at 48 h from transfection and western blot was performed to assess expression of BCL-3. Loading was assessed by blotting for Tubulin. Exemplary western blot from n=3 independent experiments.

**Supplementary Figure 5**

**
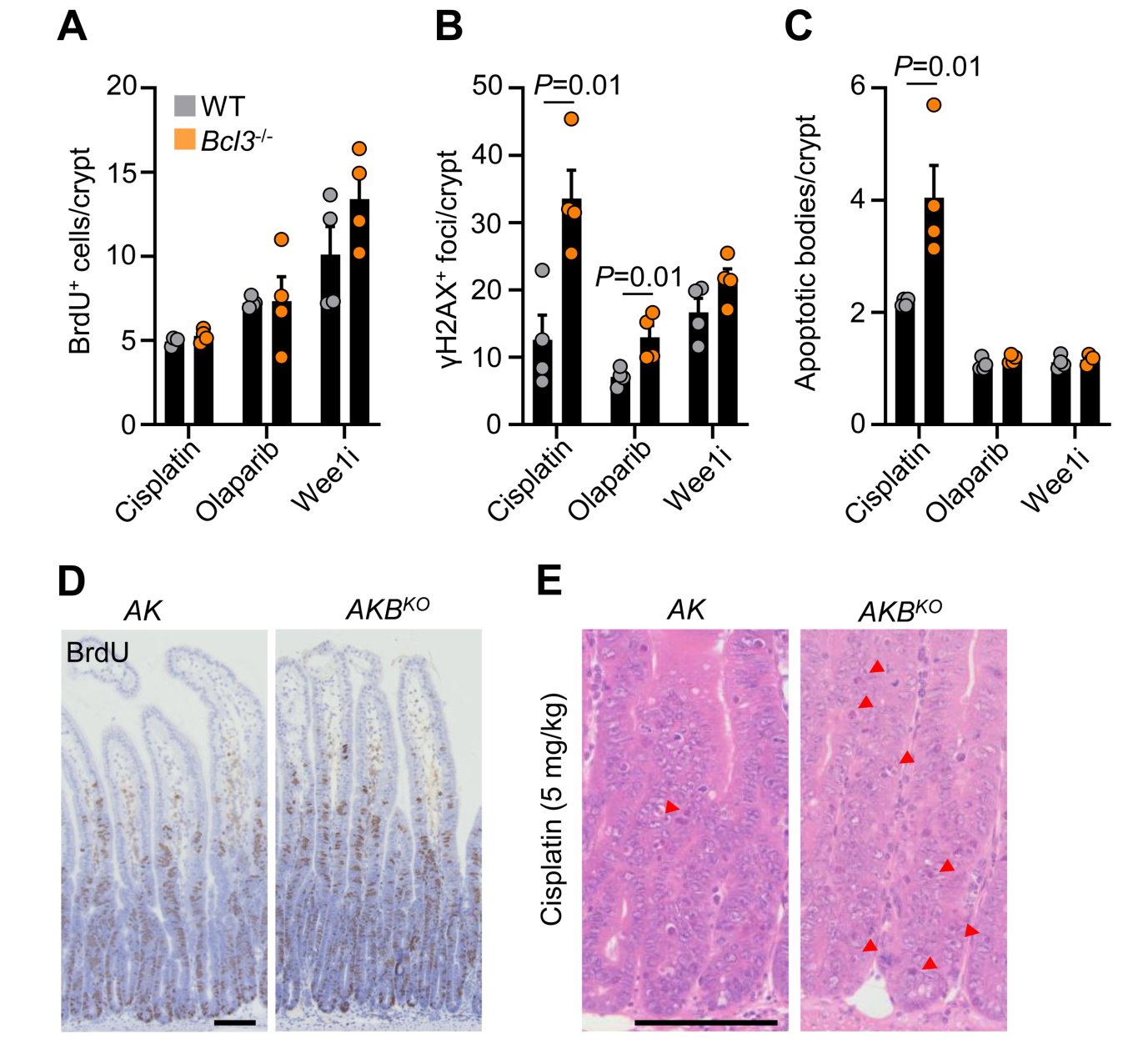
**

**Supplementary Figure 5. Bcl3 is required to protect the intestinal epithelium against DNA damage.**

**A-C.** Quantification of BrdU^+^ (**A**)**,** γH2AX^+^ (**B**) and apoptotic cells (**C**) per crypt in mice of the indicated genotype 24h after the last dose of the indicated treatments (x-axis). n=4 WT, n=4 *Bcl3*^-/-^. Data are ± s.e.m; Mann–Whitney two-tailed U-test. **D**. Representative BrdU staining of *VilCre^ER^;Apc*^fl/fl^*;Kras*^G12D/+^ (AK) and *VilCre^ER^;Apc*^fl/fl^*;Kras*^G12D/+^;*Bcl3*^-/-^ (AKB^KO^) mice 3 days post tamoxifen-induction. Scale bar, 100 μm. **E**. Representative H&E staining of tamoxifen-induced *VilCre^ER^;Apc*^fl/fl^*;Kras*^G12D/+^ (AK) and *VilCre^ER^;Apc*^fl/fl^*;Kras*^G12D/+^;*Bcl3*^-/-^ (AKB^KO^) mice 24 h post cisplatin treatment (5 mg/kg). Red arrowheads indicate apoptotic cells. Scale bar, 100 μm.
